# Prognostic value of a new score using serum alkaline phosphatase and pleural effusion lactate dehydrogenase for patients with malignant pleural effusion

**DOI:** 10.1111/1759-7714.13262

**Published:** 2019-12-13

**Authors:** Xin‐Yu Shi, Feng‐Shuang Yi, Zheng Wang, Xin Qiao, Kan Zhai

**Affiliations:** ^1^ Department of Respiratory and Critical Care Medicine Beijing Institute of Respiratory Medicine and Beijing Chao‐Yang Hospital, Capital Medical University Beijing China; ^2^ Department of Medical Research Center Beijing Institute of Respiratory Medicine and Beijing Chao‐Yang Hospital, Capital Medical University Beijing China

**Keywords:** Enzyme, malignant pleural effusion, prognosis

## Abstract

**Background:**

The objective of our study was to analyze the prognostic value of the combination of serum ALP and pleural effusion LDH (AL score) for malignant pleural effusion (MPE) patients.

**Methods:**

This study includes retrospective, descriptive and observational research from 1 June 2006 to 1 December 2017, which aimed to identify prognostic factors related to MPE patients. We analyzed the association of various clinical features, routinely tested markers from peripheral blood and MPE at diagnosis and overall survival (OS). All MPE patients were assigned to three groups according to their AL score. The impact of the AL score and other prognostic factors were evaluated with multivariable regression.

**Results:**

According to their AL score, 193 patients were assigned to three groups with 25 in group 0 (sALP < 65 U/L and pLDH < 155 U/L), 121 in group 1 (sALP > 65 U/L or pLDH > 155 U/L) and 47 (sALP > 65 U/L and pLDH > 155 U/L) in group 2. For groups 0, 1 and 2, median survival times (MST) were 23, 15 and 7 months, respectively. Among the three groups, MST, serum albumin level, C reactive protein, erythrocyte sedimentation rate, the ratios of platelet‐to‐lymphocyte, neutrophil‐to‐lymphocyte showed significant differences. The counts of neutrophils, monocytes, platelets and AL score (0 vs. 1, *P* = 0.038, hazard ratio [HR]: 1.858, 95% confidence interval [CI]: [1.034, 3.339]; 0 vs. 2, *P* = 0.001, HR: 2.993, 95% CI: [1.556, 5.531]) were independent prognostic indicators for OS of MPE patients.

**Conclusion:**

AL score is a promising indicator which can be used to predict the prognosis of MPE patients. It can assist physicians in the selection of patients for appropriate palliative treatment.

**Key points:**

To our knowledge, this paper is the first study that combined two enzymes (sALP and pLDH) from serum and pleural effusion and studied the prognostic value for MPE patients. It has been proved to be a promising indicator to assist physicians select patients for appropriate palliative treatment.

## Introduction

Most cancers can be complicated with malignant pleural effusion (MPE)^1^ and it is reported that 15% of patients with lung cancer have MPE at diagnosis which implies systemic metastasis of cancer.^2^ The life quality of MPE patients is usually compromised because of distressing symptoms, such as coughing, dyspnea, and chest pain.^3^, ^4^ It has been reported that LENT prognostic score system (pleural fluid lactate dehydrogenase, Eastern Cooperative Oncology Group performance score, neutrophil‐to‐lymphocyte ratio and tumor type) and some molecular markers could be used to predict survival of MPE patients.^3^ In view of the cost of treatment for MPE and its potential complications, it is suggested that cost‐effective and reliable prognostic indicators that might assist physicians in the precise prediction of survival time for MPE patients should be found.

Nowadays, more and more attention is being paid to the prognostic value of the change of several enzymes in cancer patients. The abnormal synthesis of enzymes exists in the process of the transformation from normal to cancer cells and tumor proliferation.^5^ Some indicators routinely tested in clinical practice, such as alkaline phosphatase (ALP), adenosine deaminase (ADA)^6^ and lactate dehydrogenase (LDH)^7^ have been investigated to determine their prognostic values in various malignancies.

ALP is an enzyme which is particularly concentrated in the liver, kidney, and bone. Researchers report that the elevation of serum ALP level is not only associated with liver diseases, but is also an indicator of poor outcome in many kinds of cancers, including esophageal cancer,^8^ colorectal cancer^9^ and prostate cancer.^10^ LDH is a ubiquitous cellular enzyme, which arises as a result of nonspecific tissue injury.^11^, ^12^ It has been described that upregulation of the LDH level can be seen in cancer, closely associated with the “Warburg effect”, which is the preferential use of glycolysis over oxidative phosphorylation in tumor cells.^11^, ^13^

The markers mentioned above from blood or MPE are obtained by routine detection with the advantage of rapid, inexpensive, and convenience in clinical practice. Previous studies have evaluated the prognostic value of serum ALP (sALP) in various cancers^14^ and pleural LDH (pLDH) has been reported to be used as a prognostic marker in MPE patients,^7^ but the combination of the two markers has not previously been studied. The objective of the current study was to investigate the prognostic value of the combination of serum ALP and pleural effusion LDH (AL score) in patients with MPE by assessing its predictability for overall survival.

## Methods

### Study population

We retrospectively reviewed all patients diagnosed with MPE at Beijing Chao‐Yang Hospital from 1 June 2006 to 1 December 2017. All diagnoses were confirmed by pleural fluid cytology and/or pleural biopsy. The patients were selected according to the inclusion criteria as follows: (i) The results of cytology or histology confirmed the diagnosis of malignant pleural effusion; (ii) untreated with anticancer therapy (including chemotherapy, radiotherapy, surgery and targeted treatment); (iii) all clinical data were available. Patients who were treated with anticoagulation therapy, or had autoimmune disease or significant infection at diagnosis were excluded from the study. This protocol was approved by the Institutional Review Board of Beijing Chao‐Yang Hospital.

### Clinical and laboratory data collection

The clinical and laboratory data were collected from medical records. Clinical and laboratory variables including age, gender, Eastern Cooperative Oncology Group performance status (ECOG PS), smoking status, distribution of pleural effusion (unilateral or bilateral), and serum levels of hemoglobin (Hb), mean platelet volume (MPV), albumin, C reactive protein (CRP), LDH, ALP, fibrinogen, D‐dimer, calcium and the counts of white blood cells (WBC), lymphocyte, eosinophils from peripheral blood at diagnosis were collected. Pleural effusion levels of total protein (TP), total cell number (TCM), LDH, ADA and the proportion of mononuclear and multinuclear cells were also collected. We also collected the tumor related variables consisting of tumor histology and tumor biomarkers such as carcino‐embryonic antigen (CEA), neuron specific enolase (NSE), squamous cell carcinoma antigen (SCC) and cytokeratin 19 fragment (CYFRA).

The ratios of neutrophil‐to‐lymphocyte (NLR) and platelet‐to‐lymphocyte (PLR) were calculated by dividing the serum counts of neutrophils and platelets by the count of lymphocytes respectively. The lymphocyte‐to‐monocyte ratio (LMR) was obtained by dividing the serum absolute count of lymphocytes by the count of monocytes.

### Defining cutoff values

The cutoff value of age was determined as median. For other clinical and laboratory parameters entered into univariate analysis on OS, maximally selected rank statistics and R software (version 3.03) were used to determine their optimal cutoff values.^15^, ^16^ The cutoff values of sALP and pLDH were defined as 65 U/L and 155 U/L, respectively.

### Categorization by AL score

According to the cutoff values of sALP and pLDH obtained by the above methods, the patients were assigned to three groups. If patients had a high sALP (>65 U/L) and high pLDH (>155 U/L) at the same time, they were assigned to group 2 (a score of 2). Patients with only one elevated parameter were assigned to group 1 (a score of 1). The remainder of the patients were assigned to group 0 (a score of 0).

### Statistical analysis

We used the SPSS statistical software package version 23.0 (SPSS, Chicago, IL, USA) and R software to perform the statistical analysis. All statistical tests were two‐sided and variables with *P*‐value < 0.05 were considered statistically significant. For continuous variables, one‐way ANOVA (analysis of variance) or Mann‐Whitney U test and χ2 test was used for comparisons of categorical data. The outcome in this study was the time from diagnosis to death or last contact. Kaplan‐Meier curves with log‐rank were used to estimate survival and compare curves when necessary. Cox regression was used to determine the prognostic values of the markers in MPE patients and the results were presented as hazards ratio (HR) and 95% confidential interval (CI).

## Results

### Patient characteristics

**Figure 1:**
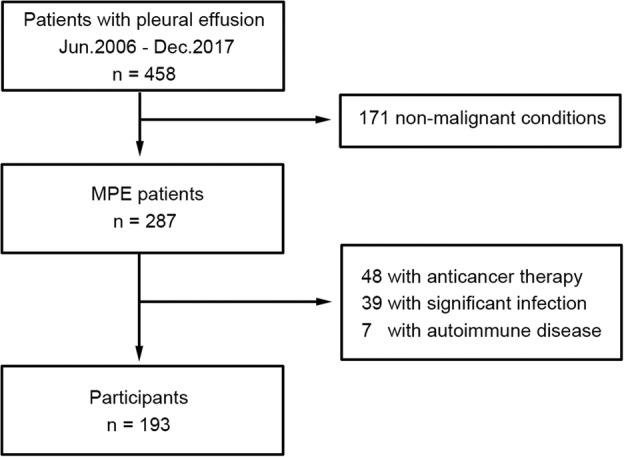
Flowchart demonstrating the process of identifying suitable patients for inclusion.

During the study period, 458 patients suffered from pleural effusion, but 171 patients were excluded from the study because of benign conditions. Among the 287 patients eligible for inclusion, 94 were eventually excluded for the reasons outlined in Figure 1. The baseline characteristics are summarized in Table 1. In total, 193 patients underwent diagnostic thoracentesis or medical thoracoscopy and the diagnosis was confirmed by cytology or biopsy. Most patients were followed‐up until death. In the current study, 145 patients developed the condition and 48 were censored. Among the 48 patients, 19 were still alive at the end of data collection and 29 were lost to follow‐up.

**Table 1:** Patient characteristics according to AL score

		AL score			
Characteristic	All patients (*n* = 193)	0 (*n* = 25)	1 (*n* = 121)	2 (*n* = 47)	*P*‐value
Median survival (month, IQR)	13 (6, 23)	23 (9, 34)	15 (7, 22)	7.0 (4.4, 9.6)	0.006*
Age (years), Median (IQR)	65 (55,73)	57 (48, 70)	64 (54, 71)	69 (59,77)	0.009*
Sex, n (%)	–	–	–	–	0.104
Male	99 (51.3)	10 (40.0)	59 (48.8)	30 (63.8)	–
Female	94 (48.7)	15 (60.0)	62 (51.2)	17 (36.2)	–
ECOG PS, n (%)	–	–	–	–	0.740
0–2	106 (54.9)	9 (36.0)	71 (58.7)	26 (55.3)	–
3–4	87 (45.1)	16 (64.0)	50 (41.3)	21 (44.7)	–
Smoking status, n (%)	–	–	–	–	0.574
Ever/current	78 (40.4)	9 (36.0)	47 (38.8)	22 (46.8)	–
Never	115 (59.6)	16 (64.0)	74 (61.2)	25 (53.2)	–
Histology, n (%)	–	–	–	–	0.943
ADC	117 (60.6)	10 (40.0)	82 (67.8)	25 (53.2)	–
SQC	10 (5.2)	1 (4.0)	6 (5.0)	3 (6.4)	–
SCLC	10 (5.2)	2 (8.0)	6 (5.0)	2 (4.3)	–
Mesothelioma	12 (6.2)	2 (8.0)	8 (6.6)	2 (4.3)	–
Others	44 (22.8)	10 (40.0)	19 (15.7)	15 (31.8)	–
EP metastasis,	–	–	–	–	–
n (%)	–	–	–	–	0.040*
Yes	105 (54.4)	17 (68.0)	58 (48.0)	31 (66.0)	–
No	88 (45.6)	8 (32.0)	63 (52.0)	16 (34.0)	–
WBC × 10^9^/L	6.82 (5.70,8.10)	6.82 (5.77, 7.17)	6.70 (5.61, 7.97)	7.50 (6.08, 8.70)	0.013*
Hb g/L	129.55 ± 17.54	128.68 ± 16.54	130.26 ± 17.42	128.19 ± 18.60	0.764
PLT × 10^9^/L	257 (211, 316)	233 (179, 3197)	279 (217, 321)	237 (211, 291)	0.330
ALB g/L	34.11 ± 4.55	36.41 ± 4.09	34.12 ± 4.46	32.87 ± 4.61	0.006*
LDH U/L	193 (165, 237)	194 (151, 214)	193 (167, 239)	193 (164, 2403)	0.326
ALP U/L	88 (70, 111)	61 (52, 64)	90 (76, 111)	95 (82, 118)	<0.001*
CRP mg/dL	1.27 (0.54, 2.90)	0.69 (0.37, 1.77)	1.09 (0.50, 2.13)	2.87 (1.48, 5.78)	<0.001*
ESR mm/hour	19 (9,30)	15 (8,26)	18 (8, 26)	25 (16, 42)	<0.001*
CEA ng/mL	5.43 (2.00, 29.46)	1.91 (1.07, 10.39)	7.23 (2.01, 35.17)	5 (2.25, 18.33)	0.328
SCC ng/mL	0.75 (0.50, 1.29)	0.70 (0.55, 0.95)	0.70 (0.50, 1.30)	0.80 (0.52, 1.48)	0.952
NSE ng/mL	16.40 (13.31, 23.54)	13.73 (11.46, 20.46)	17.53 (13.76, 23.82)	15.95 (13.92, 26.23)	0.645
CYFRA ng/mL	5.08 (2.73, 10.62)	3.16 (1.92, 6.00)	5.09 (2.74, 10.63)	6.60 (3.72, 10.63)	0.431
NLR	3.16 (2.08, 4.44)	2.10 (1.46, 3.27)	2.61 (1.87, 3.48)	5.11 (4.52, 6.66)	<0.001*
LMR	3.15 (2.24, 4.56)	4.70 (2.94, 5.83)	3.60 (2.65, 4.64)	1.96 (1.45, 2.49)	0.049*
PLR	181.63 (130.97, 245.10)	114.25 (90.23, 217.51)	162.58 (129.40, 211.64)	252.35 (198.61, 288.24)	<0.001*
pLDH U/L	250 (115, 590)	91 (58, 110)	162 (120, 596)	314 (169, 622)	0.070
pADA U/L	13 (9, 17)	13 (8, 17)	14 (9, 19)	13 (10, 16)	0.394
pTP g/L	47 (43, 52)	48 (44, 54)	47 (43, 52)	46 (42, 50)	0.318

*Statistically significant among the three groups. ADC, adenocarcinoma; AL, combination of serum ALP and pleural effusion LDH; ALB, albumin; ALP, alkaline phosphatase; CEA, carcinoembryonic antigen; CRP C, reactive protein; CYFRA, Cytokeratin 19 fragment; ECOG PS, Eastern Cooperative Oncology Group performance status; EP, extra pleural; ESR, erythrocyte sedimentation rate; HB, hemoglobin; IQR, interquartile range; LDH, lactate dehydrogenase; LMR, lymphocyte‐to‐monocyte ratio; NLR, neutrophil‐to‐lymphocyte ratio; NSE, neuron specific enolase; pADA, pleural effusion ADA; pLDH, pleural effusion LDH; PLR, platelet‐to‐lymphocyte ratio; PLT, platelet; pTP, pleural effusion total protein; SCC, Squamous cell carcinoma antigen; SCLC, small cell carcinoma; SQC, squamous cell carcinoma.

The median age was 65 years old, range 21–88. A total of 99 (51.3%) patients were male; 40.4% of patients were current or former smokers and 54.9% of patients had an ECOG PS of 0–2. Patients with adenocarcinoma accounted for 60.6%. Survival information was collected from patients' medical records and the patients were followed‐up by telephone.

### Clinical characteristics related with the new AL score

According to the score of AL, all patients were classified into three groups: 25 (12.8%) were assigned to group 0; 121 (61.1%) were in group 1; and 47 (26.1%) were in group 2. Table 1 indicates the clinical and laboratory variables associated with AL score. The medians and interquartile range (IQR) of sALP and pLDH were 88 (70, 111), 250 (115, 590), respectively. Among the three groups, gender, smoking status, ECOG PS, histology, and serum level of Hb, LDH, CEA, SCC, NSE, and pleural effusion level of ADA, and TP showed no significant difference. However, median survival time (MST, *P* = 0.006), age (*P* = 0.009), extra pleural metastasis (*P* = 0.040), WBC (*P* = 0.013), albumin (*P* = 0.006), CRP (*P* < 0.001), CYFRA (*P* = 0.431), ESR (*P* < 0.001), NLR (*P* < 0.001), PLR (*P* < 0.001), and LMR (*P* = 0.049) had significant differences among the three groups.

### AL score and overall survival

The median survival time of all patients was 13 months. Table 2 shows the results of the univariate analyses of clinical and laboratory characteristics. The variables related with shorter survival time are listed as follows: small cell lung cancer (*P* = 0.037), WBC > 3.83 × 10^9^/L (*P =* 0.003), neutrophil count (N) > 3.69 × 10^9^/L (*P* < 0.001), monocyte count (M) > 0.37 × 10^9^/L (*P* = 0.002), NLR > 4.17 (*P* = 0.001), LMR ≤ 5.71 (*P* = 0.013), fibrinogen >440.2 mg/dL (*P* = 0.042), ALP > 65 U/L (*P* = 0.009), CRP > 0.84 mg/dL (*P* = 0.001), ESR > 11 mm/hour (*P* = 0.016), CEA > 3.49 ng/mL (*P* = 0.010), NSE > 28.9 ng/mL (*P* < 0.001), pLDH > 155 U/L (*P* = 0.012), pleural effusion TP > 53 g/L (*P* = 0.003). The AL score was associated with the prognosis of MPE patients (MST of group 0 vs. 1 vs. 2, 23 vs. 15 vs. 7 months, *P* = 0.006). In the multivariable Cox model, all variables which had significant differences in the univariate analyses were included. Although age was not statistically significant in the univariate analysis, it was also included in the multivariate analysis due to clinical professional considerations. Table 2 shows the variables which were independent prognostic predictors of shorter OS in a multivariate analysis: N > 3.69 × 10^9^/L (*P* = 0.030), M > 0.37 × 10^9^/L (*P* = 0.039), PLT ≤356 × 10^9^/L (*P* = 0.013), and the increase of the AL score (*P* = 0.001).

**Table 2:** Univariate and multivariate analyses of overall survival (OS)

	Univariate analysis			Multivariate analysis		
Variable	HR	95%CI	*P*‐value	HR	95%CI	*P*‐value
Age, years	–	–	–	–	–	–
≤65	Reference	–	–	–	–	–
>65	1.095	0.792，1.516	0.582	–	–	–
Sex	–	–	–	–	–	–
Female	Reference	–	–	–	–	–
Male	1.254	0.906，1.735	0.172	–	–	–
ECOG PS	–	–	–	–	–	–
0–2	Reference	–	–	–	–	–
3–4	1.368	0.985，1.900	0.061	–	–	–
Smoking habit	–	–	–	–	–	–
Ever/current	Reference	–	–	–	–	–
Never	0.848	0.610, 1.179	0.326	–	–	–
Histology	–	–	–	–	–	–
ADC vs.	1.047	0.752, 1.459	0.784	–	–	–
SQC vs.	1.128	0.527, 2.414	0.756	–	–	–
SCLC vs.	2.140	1.046, 4.379	0.037*	–	–	–
Mesothelioma vs.	2.049	0.957, 4.386	0.065	–	–	–
Others	1.444	0.589, 3.537	0.422	–	–	–
EP metastasis metastasis	–	–	–	–	–	–
No	Reference	–	–	–	–	–
Yes	1.309	0.946，1.810	0.104	–	–	–
WBC × 10^9^/L	–	–	–	–	–	–
≤3.83	Reference	–	–	–	–	–
>3.83	2.391	1.351，4.232	0.003*	–	–	–
N × 10^9^/L	–	–	–	–	–	–
≤3.69	Reference	–	–	–	–	–
>3.69	2.178	1.467，3.236	<0.001*	1.594	1.047，2.427	0.030*
L × 10^9^/L	–	–	–	–	–	–
≤1.32	Reference	–	–	–	–	–
>1.32	0.727	0.523，1.013	0.060	–	–	–
M × 10^9^/L	–	–	–	–	–	–
≤0.37	Reference	–	–	–	–	–
>0.37	1.922	1.284，2.877	0.002*	1.567	1.023，2.398	0.039*
Hb g/L	–	–	–	–	–	–
≤107	Reference	–	–	–	–	–
>107	0.701	0.422，1.163	0.169	–	–	–
PLT × 10^9^/L	–	–	–	–	–	–
≤356	Reference	–	–	–	–	–
>356	0.595	0.354，1.000	0.050	0.511	0.301，0.866	0.013*
NLR	–	–	–	–	–	–
≤4.17	Reference	–	–	–	–	–
>4.17	1.750	1.246，2.457	0.001*	–	–	–
LMR	–	–	–	–	–	–
≤5.71	Reference	–	–	–	–	–
>5.71	0.456	0.246，0.846	0.013*	–	–	–
PLR	–	–	–	–	–	–
≤181.36	Reference	–	–	–	–	–
>181.36	1.270	0.917，1.759	0.151	–	–	–
BNP pg./mL	–	–	–	–	–	–
≤39.58	Reference	–	–	–	–	–
>39.58	1.487	0.984，2.245	0.059	–	–	–
FIB mg/dL	–	–	–	–	–	–
≤440.2	Reference	–	–	–	–	–
>440.2	1.412	1.013，1.969	0.042*	–	–	–
ALB g/L	–	–	–	–	–	–
≤29.6	Reference	–	–	–	–	–
>29.6	0.686	0.463，1.016	0.060	–	–	–
LDH U/L	–	–	–	–	–	–
≤176	Reference	–	–	–	–	–
>176	1.270	0.897，1.797	0.178	–	–	–
ALP U/L	–	–	–	–	–	–
≤65	Reference	–	–	–	–	–
>65	1.925	1.174，3.158	0.009*	–	–	–
CRP mg/dL	–	–	–	–	–	–
≤0.84	Reference	–	–	–	–	–
>0.84	1.864	1.300，2.673	0.001*	–	–	–
ESR mm/hour	–	–	–	–	–	–
≤11	Reference	–	–	–	–	–
>11	1.584	1.090，2.302	0.016*	–	–	–
CEA ng/mL	–	–	–	–	–	–
≤3.49	Reference	–	–	–	–	–
>3.49	1.556	1.110，2.183	0.010*	–	–	–
SCC ng/mL	–	–	–	–	–	–
≤1	Reference	–	–	–	–	–
>1	1.360	0.968，1.911	0.076	–	–	–
NSE ng/mL	–	–	–	–	–	–
≤28.9	Reference	–	–	–	–	–
>28.9	2.259	1.386，3.683	0.001*	–	–	–
CYFRA ng/mL	–	–	–	–	–	–
≤2.03	Reference	–	–	–	–	–
>2.03	1.478	0.879，2.486	0.141	–	–	–
Distribution of PE	–	–	–	–	–	–
Unilateral	Reference	–	–	–	–	–
Bilateral	1.410	0.877，2.267	0.157	–	–	–
pLDH U/L	–	–	–	–	–	–
≤155	Reference	–	–	–	–	–
>155	1.954	1.160，3.290	0.012*	–	–	–
pADA U/L	–	–	–	–	–	–
≤19	Reference	–	–	–	–	–
>19	0.753	0.482，1.176	0.213	–	–	–
pTP g/L	–	–	–	–	–	–
≤53	Reference	–	–	–	–	–
>53	1.805	1.223，2.664	0.003*	–	–	–
AL score	–	–	<0.001*	–	–	0.001*
0	Reference	–	–	–	–	–
1	1.876	1.047，3.361	0.034*	1.858	1.034，3.339	0.038*
2	2.811	1.492，5.295	0.001*	2.993	1.556，5.531	0.001*

ADC, adenocarcinoma; AL, combination of serum ALP and pleural effusion LDH; ALB, albumin; ALP, alkaline phosphatase; BNP, B‐type natriuretic peptide; CAR, C‐reaction protein to albumin ratio; CEA, carcino‐embryonic antigen; CRP, C‐reaction protein; CYFRA, Cytokeratin 19 fragment; ECOG PS, Eastern Cooperative Oncology Group performance status; EP, extra pleural; ESR, erythrocyte sedimentation rate; FIB, fibrinogen; Hb, hemoglobin; L, lymphocyte; LDH, lactic dehydrogenase; LMR, lymphocyte to monocyte ratio; N, neutrophil; NLR, neutrophil to lymphocyte ratio; NSE, neuron specific enolase; pADA, adenosine deaminase in pleural effusion; PLR, platelet to lymphocyte ratio; PLT, platelet; pTP, total protein in pleural effusion; SCC, squamous cell carcinoma antigen; SCLC, small cell lung cancer; SQC, squamous cell carcinoma; WBC, white blood cell.

* Statistically significant prognostic factor identified by univariate/multivariate analysis.

**Figure 2:**
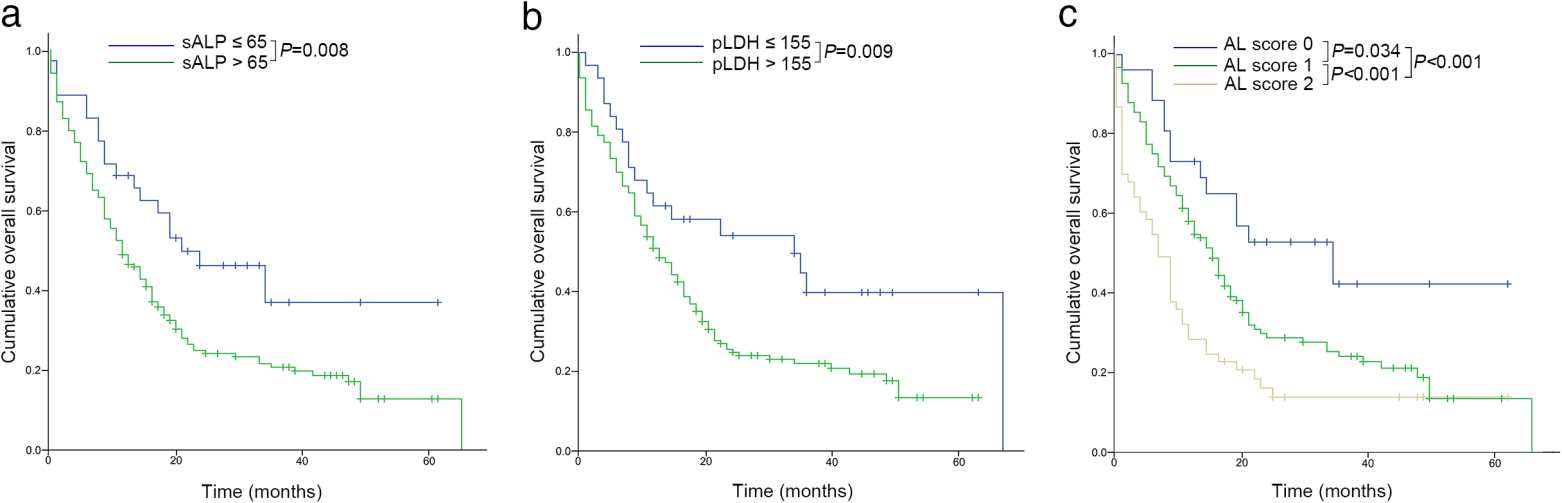
Overall survival of the total study population according to (**a**) serum alkaline phosphatase (sALP), (**b**) pleural effusion lactate dehydrogenase (pLDH), (**c**) the score of combination of serum ALP and pleural LDH (AL score).

With regard to the univariate analysis results, both sALP and pLDH were prognostic indicators: high sALP and high pLDH were prognostic indicators related with a shorter OS (MST of sALP ≤ 65 vs. >65, 22 vs. 12 months, respectively, *P* = 0.008, Fig 2a; MST of pLDH ≤ 155 vs. >155, 35 vs. 13 months, respectively, *P* = 0.009, Fig 2b). An increase in the AL score indicated a shorter OS (MST of AL score 0 vs. 1 vs. 2, 23 vs. 15 vs. 7 months, *P* < 0.001, Fig 2c).

## Discussion

With the increase in the worldwide prevalence of cancer and the improvement of anticancer therapy, the life expectancy of cancer patients has been extended and the burden of malignant pleural effusion is increasing.^17^, ^18^ It is reported that 500–700 individuals per million annually suffer from malignant pleural effusion.^19^ These MPE patients are in need of dual management; one targeted at the cancer itself and the other targeted at the drainage and prevention of recurrence of MPE.^20^, ^21^, ^22^ Precise prediction of prognosis is crucial to choosing preventive or individualized treatment for different patients. For patients with MPE, sALP and pLDH are both routinely tested enzymes. In this study, we confirmed that AL score was a prognostic predictor for OS of MPE patients.

The metabolic changes of several enzymes, such as ALP^14^ and LDH,^7^ can be the result of the rapid proliferation and invasion of tumor cells, which might indicate clinical prognosis. It has been reported that the preferential use of anaerobic glycolytic pathway of tumor cells may cause the increase of conversion from pyruvate to lactate. LDH appears to be a key enzyme during this glycolytic progress. Our findings confirm the relationship between high pLDH and poor prognosis in MPE patients and indicate validity of pLDH as a predictor of survival in this population.

In clinical practice, another easily tested enzyme is serum ALP. Nilsson *et al*.^23^ reported that ALP is a tumor related antigen. Additionally, it has been previously reported that a high ALP activity in the nucleus of cancer cells is associated with the increase of cancer cell proliferation. It has been reported that the poor prognosis of many kinds of cancers, such as prostate cancer^10^ and nasopharyngeal carcinoma,^11^ is related to high serum levels of ALP. For breast cancer patients, it is suggested that a routine measurement of sALP before and after surgery as an indicator of recurrence or metastasis is carried out.^24^

In the high AL score group, inflammation‐related markers such as NLR, PLR and the number of neutrophils, were higher than in the low score group, which means AL score has a close relationship with inflammation and immune activity. Inflammation is a known major driver for the development of cancer and inflammatory cells play a vital role in tumor microenvironment. The tumor microenvironment is thought to be an indispensable participant in tumor development, fostering proliferation, tumor survival and migration.^25^ Numerous studies have shown that neutrophils, platelets, monocytes and some cytokines, such as interleukin (IL)‐1, IL‐6, play a significant role in tumor progression.^26^ Recently, various research studies have assessed various inflammation‐related indicators to predict the prognosis of cancer patients, such as PLR,^27^ NLR,^7^ LMR.^28^ Although in the current study, these inflammation‐related indicators are independent prognostic factors, the effect of AL score on prognosis prediction is much better than the inflammation‐related markers.

To our knowledge, this paper is the first study that has combined two enzymes (sALP and pLDH) from serum and pleural effusion and studied the prognostic value of AL score in MPE patients. However, there are still some limitations. First, the size of the sample was relatively small and the study was performed in a single center, which means a large sized multi‐center study is required in the future. In the current study, all patients were diagnosed by pleural fluid cytology or biopsy of pleura, which is the critical cause of the relevant small size of the sample. Second, this study was retrospective and it therefore seems inevitable that there would be a selection, exclusion, and recall related bias. Third, this study only included routinely tested markers from serum and pleural effusion, while some molecular biomarkers (not routine tests at Beijing Chao‐Yang Hospital) described to have prognostic value for cancer patients were not considered in our study. Finally, further studies focused on elucidating the underlying mechanisms are needed.

In total, both pLDH and sALP are markers associated with tumor development, proliferation and progression, and researchers have confirmed their values as prognostic markers in various cancers. When combined, we found that AL could serve as a promising prognostic indicator for patients with MPE which is better than other parameters. We suggest that it is a useful, rapid, simple and cheap prognostic indicator for MPE patients. In addition, the results of this study should be the cornerstone for further research on sALP, pLDH and management of MPE in the future. The results will provide valuable information for clinicians in determining the most appropriate therapeutic schemes for MPE patients.

In conclusion, AL score can be used to predict the prognosis of patients with MPE. It would assist physicians to select patients fit for appropriate palliative treatment. More studies are needed to elucidate the possible underlying mechanisms and determine novel strategies for improving the outcome of these patients.

## Disclosure

The author reports no conflicts of interest in this work.
